# Malignant Sarcomatous Transformation of Benign Giant Cell Tumor of Bone after Treatment with Denosumab Therapy: A Literature Review of Reported Cases

**DOI:** 10.7759/cureus.3792

**Published:** 2018-12-28

**Authors:** Sadiq I Alaqaili, Abbas M Abduljabbar, Ali J Altaho, Abdulrahman A Khan, Jawaher A Alherabi

**Affiliations:** 1 Miscellaneous, Alfaisal University, Riyadh, SAU; 2 Emergency Medicine, Dammam University, Qatif, SAU; 3 Neurology, King Faisal University, Alahsa, SAU; 4 Orthopaedics, Alfaisal University, Riyadh, SAU

**Keywords:** giant cell tumor of bone, malignant, sarcoma, transformation, denosumab, receptor activator of nuclear factor kappa b

## Abstract

Giant cell tumor of bone (GCTB) is a biologically benign and locally aggressive tumor that most often affects the epiphyseal and metaphyseal sites of long bones in the young adult population. Overexpression of receptor activator of nuclear factor kappa B ligand (RANKL) by cancerous mesenchymal stromal cells stimulates a signal transduction cascade that recruits and activates multinucleated osteoclast-like giant cells, resulting in pathologic bone resorption. Denosumab, an RANKL inhibitor that blocks the RANKL-mediated osteoclast activation, has been recently approved by the United States Food and Drug Administration (FDA) for the treatment of aggressive GCTB. Although uncommon, several studies reported drug-related malignant morphological transformation of benign GCTB following treatment with denosumab therapy. The aim of the article was to review the clinicopathological characteristics of all the reported cases of malignant sarcomatous transformation of GCTB after treatment with denosumab therapy in patients without any history of prior exposure to radiotherapy.

## Introduction and background

Giant cell tumor of bone (GCTB) is a benign osteolytic neoplasm that primarily involves the epiphyseal and metaphyseal regions of long bones [1]. Although biologically benign, GCTB is characterized by local aggressiveness and destructive invasion into the neighboring soft tissues [2]. Around 80% to 85% of GCTB patients present to clinics with primary locally advanced neoplasms that are not amenable to surgical management [3].

The optimal management of GCTB continues to be controversial [4]. Nonetheless, surgery is the preferred standard of care as it offers curative treatment whenever technically feasible [3]. Surgical treatment choices comprise intralesional curettage and en bloc resection [3]. Surgery is generally associated with variable degrees of functional morbidity. Moreover, the rates of local recurrence are highly dependent on the type of surgery and tumor characteristics (for example, tumor site and size) [5].

From a histological point of view, GCTB has a distinctive bi-phenotypic cell pathology. GCTB comprises osteoclast-like multinucleated giant cells that express receptor activator of nuclear factor kappa B (RANK) and neoplastic mesenchymal stromal cells that express RANK ligand (RANKL). In patients with GCTB, the RANK–RANKL interaction mediates the differentiation and activation of osteoclasts and thus results in the osteolytic phenotype of bone destruction [3,6-8].

Denosumab is a fully humanized monoclonal antibody that targets RANKL. Thus, denosumab inhibits the RANK–RANKL interaction and prevents bone destruction [9]. In mid-2013, denosumab was green-lighted by the United States Food and Drug Administration (FDA) for the treatment of adults and skeletally mature adolescents with GCTB that is inoperable, or initial surgery is expected to yield considerable morbidity [3,9-10].

Several studies including clinical trials [3,10-12], case series [13-20] and case reports [21-24] have shown beneficial advantages of the utility of preoperative denosumab in the management of GCTB. Such beneficial advantages included delayed tumor progression, tumor downstaging, reduced surgical morbidity, facilitation of salvageable surgery and achievement of pre-defined therapeutic clinical/radiological/histological responses. Moreover, little is known about the local recurrence rate of GCTB following preoperative denosumab and successive intralesional curettage [20].

Prior exposure to radiation therapy has been proposed as a predisposing factor for the development of malignant transformation of GCTB [3]. Although extremely uncommon, true spontaneous malignant transformation of GCTB without prior exposure to radiation therapy has been reported in the literature [25-28]. Moreover, in patients without prior exposure to radiation therapy, several studies documented cases of malignant sarcomatous transformation of GCTB during or after denosumab therapy [29]. This has led to raising an alarming concern about a possible causal relationship between the malignant sarcomatous transformation of GCTB and denosumab therapy. However, to the best of knowledge, no article has previously summarized all the related reported cases. Therefore, the aim of this article was to provide a retrospective and narrative review of all the cases of malignant sarcomatous transformation of GCTB during or after denosumab therapy in patients without prior exposure to radiation therapy.

## Review

PubMed® database was searched until 15th December 2018 using the following keywords: “giant cell tumor of bone”, “denosumab”, and “malignant transformation”. Only English-published literature was included. Further references from published articles were also manually screened for potential additional studies. The study inclusion criteria included patients who were (i) initially diagnosed with benign GCTB, (ii) received denosumab therapy, (iii) developed malignant sarcomatous transformation during or post-therapy, and (iv) did not have any history of previous exposure to radiation therapy. For each reported case of sarcomatous transformation of GCTB during or after denosumab therapy, the following details were reported (whenever available): authors, year of publication, patient age, patient gender, tumor site, sarcoma histology, pulmonary metastases, causal relationship with denosumab therapy, time of sarcomatous transformation from the time of denosumab therapy, and duration of denosumab therapy.

In 2010, Thomas et al. [3] reported two patients who developed a sarcomatous transformation of GCTB. The first patient developed a high-grade sarcoma in the upper extremity during preoperative denosumab therapy. The second patient developed a metastatic malignant GCTB to the lung that was diagnosed eight months after cessation of denosumab therapy. The duration of denosumab therapy ranged from three to seven months. The authors stated that none of these two cases of sarcomatous transformation of GCTB were related to denosumab therapy.

In 2013, Chawla and colleagues [10] reported two patients who developed sarcomatous transformation of GCTB during preoperative denosumab therapy. In the first patient, the sarcoma was assumed to be present at the time of initial clinical presentation and probably was missed during the initial core biopsy sampling. In the second patient, the sarcoma was assumed to be a true malignant transformation of benign GCTB.

In 2015, Rutkowski and associates [11] reported two patients who developed sarcomatous transformation of GCTB in the pelvis and sacrum during preoperative denosumab therapy. The duration of preoperative denosumab therapy was roughly nine months. However, the authors indicated that none of these two cases of sarcomatous transformation of GCTB were related to denosumab therapy. Moreover, the authors pinpointed that a diagnosis of primary malignant GCTB might have been missed by sampling error at the time of initial core biopsy.

In 2015, Aponte-Tinao and partners [30] reported a 19-year-old female who developed a high-grade pleomorphic sarcoma in the proximal tibia while receiving preoperative denosumab therapy. The malignant sarcomatous transformation of GCTB originated from a recurrent benign GCTB lesion in the same initial site of the proximal tibia. The duration of preoperative denosumab therapy was 13 months. The sarcomatous transformation of GCTB was diagnosed at 13 months of denosumab therapy when a suspicious mass was clinically palpated in the popliteal fossa. The authors related the malignant sarcomatous transformation of GCTB to denosumab therapy.

In 2015, Broehm and friends [31] reported two patients who developed a sarcomatous transformation of GCTB. The first patient was a 59-year-old male with a 12-year history of GCTB and multiple recurrences. The patient developed a high-grade spindle cell osteosarcoma in the initial site (ischium) while receiving preoperative denosumab therapy. The duration of preoperative denosumab therapy was 30 months. The sarcomatous transformation of GCTB was diagnosed at 30 months of denosumab therapy. Metastatic nodules in the lungs were also detected on computed tomography scan. The second patient was a 56-year-old male with a seven-year history of GCTB. The patient developed a high-grade spindle cell osteosarcoma in the initial site (distal femur) while receiving preoperative denosumab therapy. The duration of preoperative denosumab therapy was six months. The authors related the malignant sarcomatous transformation of GCTB to denosumab therapy.

In 2016, Park and peers [32] reported a 30-year-old male patient who developed malignant transformation of GCTB after 20 months of denosumab therapy. The patient developed high-grade sarcoma at the site of initial lesion (pelvis) as well as pulmonary metastases. The authors related the malignant sarcomatous transformation of GCTB to denosumab therapy.

In 2017, Tsukamoto and co-workers [33] reported one patient who developed sarcomatous transformation of GCTB after treatment with preoperative denosumab. The patient was a 29-year-old woman who underwent intralesional curettage of a GCTB in the left ischium. Approximately ten years after the initial surgery, the patient developed a recurrence of GCTB in the same original site. The patient received preoperative denosumab therapy for six months. At six months, a computed tomography scan showed a rapidly growing lesion in the left ischium. A histopathological examination of the incisional biopsy was consistent with high-grade osteosarcoma. The patient progressed clinically and developed pulmonary metastases and eventually died. The authors related the malignant sarcomatous transformation of GCTB to denosumab therapy.

Table 1 shows a summary of the published studies on malignant sarcomatous transformation of benign GCTB following treatment with denosumab therapy. To the best of knowledge, 11 related cases have been reported so far in the English literature. In five cases, the authors declined a causal relationship between the malignant sarcomatous transformation of GCTB and denosumab therapy. In six cases, the authors related the malignant sarcomatous transformation of GCTB to denosumab therapy. High-grade osteosarcoma was the most common tumor histology. All cases of malignant sarcomatous transformation of GCTB occurred in the initial site of the tumor, and four patients developed malignant pulmonary metastases. The duration of denosumab therapy ranged from six to 30 months.

**Table 1:** Summary of all published studies about malignant sarcomatous transformation of giant cell tumor of bone during and after denosumab therapy GCTB: giant cell tumor of bone; NM: not mentioned; Ref: reference

Ref	Authors	Year	Patient (*n*)	Sex	Age (years)	Tumor location	Tumor histology	Pulmonary metastases	Causal relationship with denosumab therapy	Duration of denosumab therapy
[3]	Thomas et al.	2010	1	NM	NM	Upper extremity	High-grade sarcoma	NR	No	3-7 months
			1	NM	NM	NM	Malignant GCTB	Yes	No	3-7 months
[10]	Chawla et al.	2013	1	NM	NM	NM	NM	NM	No	NM
			1	NM	NM	NM	NM	NM	Yes	NM
[11]	Rutkowski et al.	2015	1	NM	NM	Pelvis	NM	NM	No	9.2 months
			1	NM	NM	Sacrum	NM	NM	No	9.2 months
[30]	Aponte-Tinao et al.	2015	1	Female	19	Proximal tibia	High-grade pleomorphic sarcoma	NM	Yes	13 months
[31]	Broehm et al.	2015	1	Male	59	Ischium	High-grade spindle cell osteosarcoma	Yes	Yes	30 months
			1	Male	56	Distal femur	High-grade spindle cell osteosarcoma	NM	Yes	6 months
[32]	Park et al.	2016	1	Male	30	Pelvis	High-grade sarcoma	Yes	Yes	20 months
[33]	Tsukamoto et al.	2017	1	Female	29	Ischium	High-grade osteosarcoma	Yes	Yes	6 months

The exact mechanism by which the sarcomatous transformation of GCTB occurs during or after denosumab therapy is poorly defined [33]. However, from a molecular point of view, three potential hypotheses are proposed. First, RANKL expression contributes substantial roles in B- and T-cell differentiation as well as dendritic cell survival [34-36]. Thus, the inhibition of RANKL expression by denosumab therapy is expected to lead to immunosuppression and subsequently increase the potential occurrence of various infectious and neoplastic processes. Second, RANKL expression upregulates nuclear factor IB (NFIB), which is associated with decreased vulnerability to various nuclear oncogenes [37-38]. Thus, the inhibition of RANKL expression by denosumab therapy is expected to promote osteosarcoma carcinogenesis by increasing predisposition to various nuclear oncogenes. Third, RANKL expression upregulates semaphorin 3A gene expression in osteosarcoma cell lines [38], and knockout of this gene stimulates deregulated bone and cartilage growth [39]. Thus, the inhibition of RANKL expression by denosumab therapy is expected to induce abnormal proliferation and differentiation of osteoblasts and bring about semaphorin 3A-mediated osteosarcoma carcinogenesis.

Furthermore, from a technical point of view, a potential hypothesis has been proposed to account for the sarcomatous transformation of GCTB that takes place during or after denosumab therapy. This hypothesis takes into account that osteosarcoma arising in a histologically benign GCTB may encompass some scattered foci of malignant tumor. These foci of malignant tumor (osteosarcoma) may have been missed by means of sampling error at the time of initial core biopsy [11,40]. Moreover, it should be noted that confounding factors (for example, age, gender, and previous treatment with selective intra-arterial embolization) may play contributing roles to the malignant sarcomatous transformation seen in GCTB patients treated with denosumab therapy. This is an interesting topic for future research exploration.

In literature, apart from the sarcomatous transformation, a wide variety of morphological transformation of GCTB has been reported during and after denosumab therapy. For instance, some authors described growth patterns resembling osteoblastoma [41], fibrous histiocytoma [42-43] and an aggressive form of osteoid osteoma that looks like osteosarcoma [44-45]. Moreover, it has been speculated that morphological transformations may be correlated with the duration of denosumab therapy [45]. For example, short-term denosumab therapy will result in early excised lesions displaying spindle-shaped cell stroma, whereas long-term denosumab therapy will result in later excised lesions displaying more dense bone disposition resembling low-grade central osteosarcoma or fibrous dysplasia. Wojcik and partners [45] pinpointed that denosumab-treated GCTB exhibit an extent of structural overlap with malignant GCTB. Thus, careful history documentation of previous denosumab administration is vital to circumvent a possible misdiagnosis of malignant GCTB.

At the present time, the optimal duration of denosumab therapy is not yet established. In addition, whether the duration of denosumab therapy is directly associated with the increased potential of developing sarcomatous transformation of GCTB remains poorly defined. Nevertheless, long-term treatment with denosumab therapy should be limited to the minimum possible in order to avoid dose-dependent toxicity side effects, for example, osteonecrosis of the jaw [46].

Lastly, it should be noted that this review article may not be reporting all the cases of malignant sarcomatous transformation of GCTB, as under-reporting of such cases by healthcare centers remains a possibility. Thus, the number of related cases reported in this review (*n *= 11) may not represent the actual figure.

## Conclusions

In conclusion, healthcare providers should be aware that malignant sarcomatous transformation of GCTB during or after denosumab therapy is a possible consequence. To the best of knowledge, 11 (*n *= 11) related cases have been reported so far in the English literature. The exact mechanisms or triggering factors by which sarcomatous transformation of GCTB occurs during or after denosumab therapy are poorly defined. Additional isolated case reports and large-sized cohort studies are needed to thoroughly explore a causal association between the malignant sarcomatous transformation of GCTB and denosumab therapy.
